# Primary angiosarcoma of the testis: report of a rare entity and review of the literature

**DOI:** 10.1186/1746-1596-2-23

**Published:** 2007-07-02

**Authors:** Henry B Armah, Uma NM Rao, Anil V Parwani

**Affiliations:** 1 Department of Pathology, University of Pittsburgh Medical Center, Pittsburgh, PA, USA

## Abstract

**Background:**

Primary testicular angiosarcomas are extremely rare, and their clinicopathologic features are not well described. Our objective was to further define the clinical features and pathologic spectra of primary testicular angiosarcomas.

**Methods:**

Six previously reported case reports were identified in the English language medical literature using MEDLINE and a subsequent bibliographic search of all pertinent reports and reviews was performed. After excluding 2 cases because they did not involve the testis, we identified 4 previously reported cases of true primary testicular angiosarcoma. We also searched the electronic medical archival records of our institution and identified one additional unreported case of true primary testicular angiosarcomas. Data were extracted on the demographics, predisposing factors, clinical presentation, gross pathology, microscopic pathology, immunophenotype, therapy, and outcomes of each of these 5 cases of true primary testicular angiosarcomas.

**Results:**

Primary testicular angiosarcomas were found at a mean age of 43.4 years. None of the cases was associated with exposure to radiation, arsenic, thorium dioxide, or vinyl chloride. However, 1 case was associated with hydrocele. It typically presented with painless mass (mean size, 6.3 cm). Histologically, all showed classic anastomosing channels lined by plump hyperchromatic cells, though most showed epithelioid cytology and some showed solid architectural pattern. One patient had multiple metastatic recurrences but eventual outcome was not available, and 1 patient died a month after diagnosis from stroke but no autopsy was performed. The remaining 3 patients were alive at the time of publication of their respective cases (mean, 17 months).

**Conclusion:**

Primary testicular angiosarcomas are typically rare tumors of men of all ages that appear to segregate into 2 groups; one associated with teratoma and occurring in young people, and the other occurring in the elderly and not associated with germ cell neoplasm, but may be associated with chronic hydrocele. They present with advanced disease and show a wide histologic spectrum. However, their prognosis may be better than previously thought.

## Background

Angiosarcomas generally occur in the skin and rarely in the deep soft tissue and viscera. Primary angiosarcoma of the testis is an extremely rare tumor. The first bona fide case of true primary testicular angiosarcoma was described in 1991 by Hughes et al [1] To our knowledge, only 6 cases, and possibly as few as 4 true primary cases, have been reported in the English language medical literature [1-6]. The biologic behavior of these tumors is not clear since they are usually categorized with deep soft tissue angiosarcomas, which in general have a higher mortality than their cutaneous counterparts [7]. The pathologic features of true primary testicular angiosarcomas are not well described, and with the rarity of these cases, a diagnosis may be extremely difficult to render, particularly on limited biopsy material. Additionally, little information exists about its natural history due to its rarity. In this article, we review the clinical and pathologic features of primary testicular angiosarcomas, focusing on the histologic spectrum of these tumors. We include a case retrieved from the electronic medical archival records of our institution (described in the tables as "current case") that has not been published previously.

## Methods

Six prior case reports of angiosarcoma of the testis were found in the English language medical literature in a MEDLINE search. Of these, 2 were excluded from the analysis because they were retroperitoneal [5] and paravertebral [6] angiosarcomas (did not involve the testis) arising 5 and 10 years after radiotherapy for testicular teratoma and seminoma, respectively. Additionally, the computerized medical archival records of the University of Pittsburgh Medical Center (1986 to present) was searched and 1 additional unreported case of testicular angiosarcoma was found. Formalin-fixed, paraffin-embedded tissue sections and the following immunohistochemical stains from this case were reviewed: Ulex Europaeus lectin (1:50, EY Lab, San Francisco, California), CD31 (1:20, DakoCytomation, Carpinteria, California), CD34 (1:20, Becton Dickinson, San Jose, California), AE1/AE3 (AE1/AE3, 1:500, Dako), and Pancytokeratin cocktail (AE1/AE3, 1:500, Dako; CAM5.2, 1:50, Becton Dickinson; MNF116, 1:50, Dako; and UCD/PR-10.11, 1:25, Zymed, San Francisco, California). Tissue sections that had been shown to be positive or negative for each marker were used as controls.

The histologic diagnosis was based on the morphologic spectra defined by Meis-Kindblom and Kindblom [7]. The architectural categories were: classic, a proliferation of anastomosing blood-filled channels; solid, a proliferation of sheets or nests without evidence of lumen formation; and primitive, a nearly solid proliferation with angulated small capillary-sized lumina reminiscent of tubules but filled with blood. The cytologic categories were: typical, plump hyperchromatic, tufted endothelial cells with scant amphophilic cytoplasm; epithelioid, endothelial cells with open chromatin, prominent nucleoli, and abundant eosinophilic cytoplasm; and spindled, fusiform or elongated cells with tapered ends.

## Results

### Clinical features

Clinical characteristics of all the reported primary testicular angiosarcomas are summarized in Table 1[1-4]. Primary testicular angiosarcomas occurred in both young and old men (range, 16 to 80 years; mean age, 43.4 years). None of the 5 cases had antecedent radiation therapy. Additionally, none had a prior history of exposure to arsenic, thorium dioxide, or vinyl chloride. However, the current case was associated with a 7-year history of hydrocele on the ipsilateral side as his testicular angiosarcoma. All the 5 cases of primary testicular angiosarcomas presented with painless testicular mass. Other signs and symptoms described included intermittent fever (20%; 1/5), flank pain (20%; 1/5), and hydrocele (20%; 1/5). Three (60%) of the 5 cases of primary testicular angiosarcoma were associated with the concurrent presence of multilocular cystic areas diagnosed as mature teratoma in the same testicular tumor [1,3,4]. An extensively sampled multilocular cystic testicular tumor showing only components of mature teratoma occurred synchronously with metastatic mature teratoma with high-grade angiosarcoma in a 10 cm solid and cystic retroperitoneal mass and enlarged retroperitoneal lymph nodes [4]. The retroperitoneal mass and lymph nodes were considered as metastasis from the testicular tumor because they contained both teratomatous and angiosarcomatous elements. Although the primary testicular tumor was adequately sampled, the possibility of a small unsampled focus of angiosarcoma could not be entirely excluded [4], and hence this case was included as a case of true primary testicular angiosarcoma.

**Table 1:** Clinical characteristics of primary testicular angiosarcoma

**Source, year**	**Age, year**	**Predisposing Factors**	**Clinical Presentation**	**Primary Diagnosis of Testicular Tumor**	**Association with Testicular Germ Cell Tumor**
Hughes et al, 1991 [1]	16	None	1-month history of painless right testicular swelling	Mature teratoma and well-differentiated angiosarcoma	Yes
Masera et al, 1999 [2]	74	None	3-week history of left testicular mass and intermittent fever	Epithelioid angiosarcoma	No
Steele et al, 2000 [3]	24	None	6-month history of left testicular mass	Mature teratoma and epithelioid angiosarcoma	Yes
Sahoo et al, 2003 [4]	23	None	Right testicular mass and severe intermittent right flank pain	Mature teratoma	Yes
Current case	80	None	2-month history of painless right testicular mass and 7-year history of right hydrocele	Angiosarcoma, not otherwise specified (NOS)	No

### Pathologic features

Gross, microscopic, and immunohistochemical features of all the reported primary testicular angiosarcomas are summarized in Table 2[1-4].

**Table 2:** Pathologic characteristics of primary testicular angiosarcoma

**Source, year**	**Macroscopic findings of angiosarcoma**						
					
	**Gross**	**Site**	**Size, cm**	**Architectural patterns of angiosarcoma**	**Cytologic features**	**Mitotic rate**	**Immuno-phenotype**
Hughes et al, 1991 [1]	*Flesh-colored intervening stroma	Testicular parenchyma	9	* Classic	Typical	NA	FVII^+^, UEL^+^
Masera et al, 1999 [2]	Well-vascularised lesion	Testicular parenchyma	1.7	Classic	Epithelioid	High	FVIII^+^, UEL^+^, CD31^+^, CD34^+^, Pancytokeratin^-^, CD68^-^, LCA^-^, PLAP^-^
Steele et al, 2000 [3]	*Vascular solid mass	Testicular parenchyma with invasion of rete testis, epididymis, and spermatic cord	8	*Classic	Epithelioid	NA	FVIII^+^, CD31^+^
Sahoo et al, 2003 [4]	#Vascular solid mass	#Retroperitoneum	10	#Classic, focally solid	Epithelioid and spindled	NA	CD31^+^, CD34^+^, AE1/AE3^-^, CAM5.2^-^, PLAP^-^
Current case	Solid vascular nodule	Testicular parenchyma with invasion of epididymis	3	Classic, focally solid	Typical	8/10 HPF	UEL^+^, CD31^+^, CD34^+^, AE1/AE3^-^, Pancytokeratin^-^

NA: not available; FVIII, factor VIII-related antigen; UEL, Ulex europaeus lectin, LCA, leucocyte common antigen; PLAP, placental alkaline phosphatase.

*Concurrent presence of multiloculated cystic areas diagnosed as mature teratoma in the same testicular tumor.

#Concurrent presence of multiloculated cystic testicular tumor diagnosed as mature teratoma.

### Gross findings

**Figure 1:**
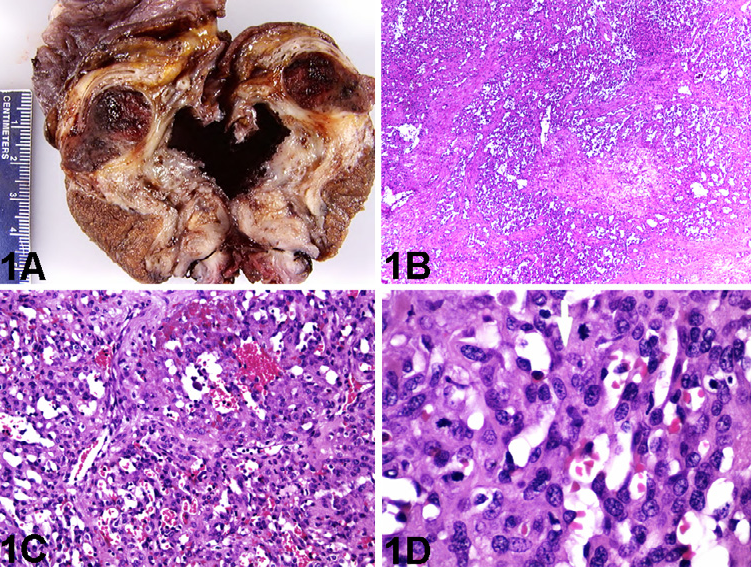
Testicular tumor. A, Sectioned surface of testicular tumor shows an expansile hemorrhagic nodule displacing surrounding testicular tissue (gross). B, Angiosarcoma with classic architectural pattern composed of proliferation of anastomosing blood-filled channels, and focal solid pattern (hematoxylin-eosin, original magnification ×40). C, Angiosarcoma with classic architectural pattern composed of anastomosing small, apparently immature vascular channels, lined by crowded plump endothelial cells, proliferating in a fibromyxoid stroma (hematoxylin-eosin, original magnification ×200). D, Tumor cells with typical cytology of plump hyperchromatic, tufted endothelial cells with scant amphophilic cytoplasm. Note the presence of mitotic figures (hematoxylin-eosin, original magnification ×600).

The size of the tumors in the 5 cases of primary testicular angiosarcomas ranged from 1.7 to 10 cm in greatest dimension (mean, 6.3 cm). On gross examination, the vast majority of the testicular angiosarcomas were solid vascular lesions (Figure 1A) [80%; 4/5], while the remaining 1 presented as flesh-colored intervening stroma in a multilocular cystic mature testicular teratoma. The site most commonly involved was the testicular parenchyma in all 5 cases, followed by local invasion of the epididymis (40%; 2/5) and the spermatic cord (20%; 1/5). Gross examination of the right orchiectomy and hydrocelectomy specimen in the current case showed a 3 cm solid vascular nodule of the testicular parenchyma and epididymis, with no tumor involvement of the spermatic cord and hydrocele sac (Figure 1A).

### Microscopic findings

All the 5 cases of primary testicular angiosarcomas showed the classic architectural pattern of proliferating anastomosing blood-filled channels (Figure 1B). At least focal solid patterns were seen in 2 (40%) of the 5 cases. Although most of these tumors showed cytologic features of epithelioid angiosarcomas (60%; 3/5), the typical cytology of plump hyperchromatic, tufted endothelial cells with scant amphophilic cytoplasm (Figures 1C &1D) was seen in 2 (40%) of the 5 cases, and the spindled cytology was seen in 1 (20%) of 5 cases. Our current case revealed proliferation of anastomosing blood-filled channels, and focal areas of proliferation of sheets or nests without evidence of lumen formation. The constituent cells were plump hyperchromatic, tufted endothelial cells with scant amphophilic cytoplasm (Figures 1C &1D). The mitotic rate of the tumor was characterized in 2 cases and only quantitatively in 1 case (the current case, 8/10 mitoses per high-power fields). The mitotic rate was characterized as high in the other case. There was no evidence of intratubular germ cell neoplasia of the unclassified type (IGCNU) in any of the 5 cases of primary testicular angiosarcomas.

### Immunohistochemical findings

**Figure 2:**
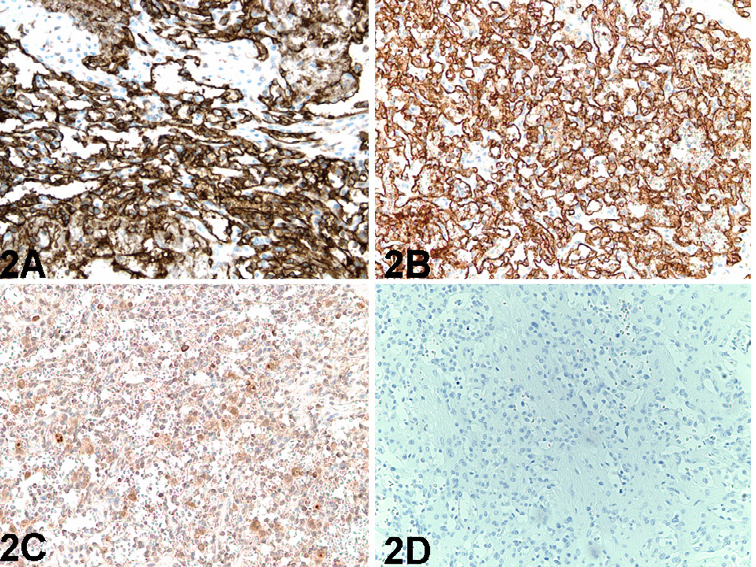
Testicular tumor. A, Immunoperoxidase staining for CD34 demonstrates strong positive reactivity in malignant endothelial cells lining the vascular channels (immunoperoxidase, original magnification ×200). B, Immunoperoxidase staining for CD31 demonstrates strong positive reactivity in malignant endothelial cells lining the vascular channels (immunoperoxidase, original magnification ×200). C, Immunoperoxidase staining for Ulex Europaeus lectin demonstrates positive reactivity in malignant endothelial cells lining the vascular channels (immunoperoxidase, original magnification ×200). D, Immunoperoxidase staining for Pancytokeratin demonstrates negative reactivity in the malignant endothelial cells lining the vascular channels (immunoperoxidase, original magnification ×200).

Immunohistochemical staining was performed in all 5 cases, but the panel used varied from case to case. Factor VIII-related antigen was positive in all the cases in which it was used (100%; 3/3). The other immunohistochemical markers that were positive in all the cases in which they were used were CD34 (Figure 2A) (100%; 3/3), CD31 (Figure 2B) (100%; 4/4), and Ulex Europaeus lectin (Figure 2C) (100%; 3/3). Immunohistochemical markers that were negative in all the cases in which they was used were Pancytokeratin (Figure 2D) (0%; 2/2), AE1/AE3 (0%; 2/2), Placental Alkaline Phosphatase (0%; 2/2), CAM5.2 (0%; 1/1), CD68 (0%; 1/1), and Leucocyte Common Antigen (0%; 1/1).

### Treatment, follow-up and prognosis

Treatment and follow-up for all reported primary testicular angiosarcomas are summarized in Table 3[1-4]. Surgery was the primary treatment in all cases. All 5 patients had orchiectomy, and margins were negative in all 5 cases. Additional treatment in the form of chemotherapy was given in 2 (40%) of 5 cases. Local recurrences were absent in all 5 cases. Lymph node metastases were seen in 2 (40%) of 5 cases; to the renal hilar and para-aortic region in 1 case, and to the retroperitoneal region in the other. Distant metastases were seen in the same 2 (40%) of 5 cases as well, with lung metastases in both cases, and additional liver, retroperitoneal and pleural implants in 1 case. Follow-up was available for all 5 cases but incomplete for 2 cases. One died a month after diagnosis and orchiectomy from stroke but no autopsy was performed to confirm that he was free of angiosarcoma at the time of death. The other case with incomplete follow-up was reported to have had multiple metastatic recurrences despite aggressive chemotherapy but his ultimate fate was not indicated as at the time of publication of his case report. The remaining 3 patients with complete follow-up were alive as of the publication of their respective cases (mean follow-up, 17 months).

**Table 3:** Treatment and follow-up for primary testicular angiosarcoma

**Source, year**	**Primary Treatment**	**Final Margins**	**Additional Treatment**	**Pattern of Spread and Progression**		**Outcome**
					
				**Local Recurrences**	**Metastases**	
Hughes et al, 1991 [1]	Orchiectomy	Negative	No	No	No	Alive and well 9 months after diagnosis
Masera et al, 1999 [2]	Orchiectomy	Negative	No	No	No	Died 1 month after diagnosis from stroke, no autopsy
Steele et al, 2000 [3]	Orchiectomy	Negative	Chemotherapy	No	Lung, renal hilar and para-aortic lymph nodes	Multiple metastatic recurrences despite aggressive chemotherapy
Sahoo et al, 2003 [4]	Orchiectomy	Negative	Chemotherapy	No	Retroperitoneum, retroperitoneal lymph nodes, liver, lung and pleura	Alive and well 22 months after diagnosis
Current case	Orchiectomy	Negative	No	No	No	Alive and well 20 months after diagnosis

## Discussion

Angiosarcomas are rare, highly aggressive tumors of vascular and lymphatic endothelial cell origin, that comprise less that 2% of all sarcomas and less than 1% of all vascular tumors[7,8]. Angiosarcomas are known to be associated with chronic lymphedema, radiotherapy, and exposure to arsenic, thorium dioxide, or vinyl chloride [7,8]. It is frequently described in upper limb lymphedema as a result of mastectomy and radiotherapy for breast carcinoma. Other commonly reported sites include breast, liver, spleen, bladder, and bone, but very rarely in the testis. Approximately 10% of the cases of vascular sarcomas are associated with postmastectomy lymphoedema [7,8]. The overall prognosis of angiosarcoma is poor, with a mean survival of 24 months and a 5-year survival of 10–24% [7-9]. Although data are limited, deep soft tissue and visceral angiosarcomas appear to have an even higher mortality, with up to 53% of patients succumbing to disease within a year [7,8]. Less than one third of deep angiosarcomas arise in visceral organs, with the liver, spleen, and heart being the most common sites of involvement [7]. The mainstay of treatment for testicular angiosarcoma is surgery. Chemotherapy is only palliative, and the value of high-dose radiotherapy is limited in radiation-induced angiosarcoma because of the tumour bed having been previously irradiated to near-normal tissue tolerance doses [10]. The reported actuarial disease-free survival was 43% when adjuvant radiotherapy is given in angiosarcomas, as compared to 17% without [8].

To our knowledge, only 4 cases of true primary testicular angiosarcomas have been previously reported in the English language medical literature [1-4]. Hence the characterization of the etiology and clinicopathologic features of primary testicular angiosarcoma is not well defined in the medical literature. Indeed, the clinical, gross, histopathologic, and immunophenotypic features of angiosarcomas of the testis have not been previously reviewed comprehensively. Initially, 6 prior case reports of angiosarcoma of the testis were found in the English language medical literature in a MEDLINE search. Of these, 2 were excluded from the analysis. These 2 were excluded because they were not true primary testicular angiosarcomas [5,6]. The case reported by Ulbright et al [5] in a 17-year old male was a retroperitoneal angiosarcoma arising 5 years after pre-operative radiotherapy of 45 Gy to the retroperitoneum for metastatic testicular mature teratoma. Of note, both the resected retroperitoneal metastasis that occurred synchronous with the testicular mass 5 years earlier were diagnosed as mature teratoma, after thorough sampling. Therefore the retroperitoneal angiosarcoma arising 5 years later was most probably de novo radiation-induced, and not arising from or a remnant of an unsampled angiosarcoma in the testicular and/or retroperitoneal mature teratoma 5 years earlier [5]. Similarly, the case reported by Lee et al [6] in a 35-year old man was a thoracic paravertebral angiosarcoma arising 10 years after prophylactic post-operative radiotherapy to the mediastinum following orchiectomy for a 8 cm stage I pure seminoma. Of note, computerized tomography scans of the chest, abdomen, and pelvis were negative at the time of diagnosis of the pure seminoma of the testis 10 years earlier. This confirmed the absence of metastasis of the seminoma to the mediastinum or para-aortic region 10 years earlier, and hence the estimated dose of 28 to 54 Gy to the mediastinum was given only prophylactically. Therefore the paravertebral angiosarcoma arising 10 years later was most probably de novo radiation-induced [6]. Thus previously published data was available on only 4 cases of true primary testicular angiosarcoma for our review.

With the inclusion of our current case, 5 cases of true primary testicular angiosarcoma formed the basis of our analysis and subsequent comments. Clinically, primary testicular angiosarcomas occurred across the age spectrum for adult men, in both young and old men (mean age, 43.4 years; respective ages were 16, 23, 24, 74, and 80). The biologic behavior and prognosis of true primary testicular angiosarcoma may be much better than previously thought. None of the 5 patients died with metastatic disease, though follow-up was incomplete for 2 cases. The remaining 3 patients with complete follow-up were alive and well at the time of their respective publications (mean follow-up, 17 months). Hence, although definitive conclusions cannot be drawn from such a small set of patients without long term 5-year follow-up, the ultimate biologic behavior of true primary testicular angiosarcomas may not be as abysmal as previously thought on comparison with visceral and deep soft tissue angiosarcomas [7-9].

Visceral angiosarcomas are conventionally regarded as deep soft tissue angiosarcomas because they typically present as hemorrhagic masses [7]. The vast majority of true primary testicular angiosarcomas presented as hemorrhagic masses, typical of visceral and deep soft tissue angiosarcomas. In a review of 80 cases involving deep soft tissues, most of these angiosarcomas were found to be heterogeneous in morphology and the most common pattern seen was the epithelioid cytologic pattern (70%) [7]. Epithelioid cytologic pattern was found in a comparable percentage (80%) of cases of primary testicular angiosarcoma in this series [2-4]. Classical architectural pattern of relatively well-differentiated areas of anastomosing blood-filled channels lined by atypical cells and infiltrating soft tissue, classic for cutaneous or mammary angiosarcomas, were observed in only 8% of their cases of deep soft tissue angiosarcomas [7]. This classic architectural pattern with typical cytology was found in 2 (40%) of the 5 cases of primary testicular angiosarcoma herein reviewed [1], although the most common (60%) pattern reported in the 5 cases of primary testicular angiosarcoma was classic architectural pattern with epithelioid cytologic features [2-4]. However, 40% of the 5 cases of primary testicular angiosarcoma had a focal solid growth component in addition to the classic architectural pattern [4] and 20% had a spindled cytology in addition to the epithelioid cytology [4].

Our further definition of the histopathologic spectrum of testicular angiosarcoma is important from a diagnostic standpoint, especially when faced with small biopsy specimens. Epithelioid cytology and solid architectural patterns are apparently not an uncommon finding in testicular angiosarcomas and may be mistaken in some cases for a poorly differentiated carcinoma, as illustrated by the case report of Sahoo et al [4] where the angiosarcoma was mistaken for an embryonal carcinoma by the primary pathologist. Other differential diagnoses of angiosarcoma include melanoma, anaplastic large cell lymphoma, other sarcomas, and other vascular lesions, particularly reactive posttherapy changes. Based on our review, the most useful histomorphologic characteristics to raise one's suspicion of an angiosarcoma is the presence of blood-filled spaces lined by atypical, often epithelioid, endothelial cells. Angiosarcomas with epithelioid cytology mimics carcinoma. Since both angiosarcomas and carcinomas can be cytokeratin positive, it is important to perform immunohistochemistry using a large panel of markers including CD31, CD34, and Ulex Europaeus lectin. For the distinction between a carcinoma and angiosarcoma in a rare location such as the testis, a single immunohistochemical stain may not be sufficient to confirm the diagnosis. Although no testicular angiosarcoma has been reported to have cytokeratin reactivity to date [2-4], overall up to one third of angiosarcomas have been reported to have cytokeratin reactivity, including those without epithelioid histology [7]. Some authors [7] suggest that Factor VIII-related antigen is the most sensitive marker in deep soft tissue and visceral angiosarcomas, including testicular angiosarcomas. In this review, testicular angiosarcomas showed positive immunoreactivity to Factor VIII-related antigen, CD31, CD34, and Ulex Europaeus lectin in all the cases in which they was used. Conversely, testicular angiosarcomas showed negative immunoreactivity to Pancytokeratin, AE1-AE3, Placental Alkaline Phosphatase, CD68, and Leucocyte Common Antigen in all the cases in which they was used. However, because of the variability among immunohistochemistry laboratories, more than one endothelial marker would be necessary to support the diagnosis of angiosarcoma in a site such as the testis where the entity is extremely rare.

The known angiosarcoma risk factors of antecedent radiation therapy, and prior exposure to arsenic, thorium dioxide, or vinyl chloride [7-10] were absent in all 5 cases of true primary testicular angiosarcoma herein reviewed. However, the associated ipsilateral chronic hydrocele in our current case may be a risk factor for the development of primary testicular angiosarcoma, though this association has not been previously reported. Chronic lymphedema, specifically scrotal edema and postmastectomy lymphedema of upper limb, are risk factors for the development of angiosarcoma of the scrotum and upper limb, respectively [7,8]. Approximately 10% of the cases of vascular sarcomas are associated with postmastectomy lymphoedema [7,8]. Hydrocele is the most common cause of scrotal swelling [11]. It is a common, benign condition manifesting as fluid collection between the wall layers of the tunica vaginalis. It usually results from a patent processus vaginalis that communicates with the peritoneal cavity. Less frequently, a non-communicating hydrocele can be found in the inguinal canal or the scrotum secondary to tumor, trauma, inflammation or infection [11]. Post-traumatic hydroceles are seen mainly in older men, as was the case in our current 80-year-old patient whose primary testicular angiosarcoma did not arise from a teratoma. Although an association has not been reported between testicular angiosarcoma and hydrocele, other testicular and paratesticular sarcomas (namely leiomyosarcomas, rhabdomyosarcomas, and osteosarcomas) have been associated with hydrocele [12-16]. Williams and Banerjee reviewed 13 cases of leiomyosarcoma in the scrotum, 3 of which were associated with hydrocele, and suggested that the presence of hydrocele in association with paratesticular tumor is usually an indication that the tumor is malignant [15]. Hence, the ipsilateral chronic hydrocele may be a risk factor for the development of the testicular angiosarcoma in the current case. Although it is well recognized that hydroceles may also develop secondarily to the presence of a testicular mass, the 7-year history of hydrocele prior to the discovery of the testicular mass and the diagnosis of the testicular angiosarcoma makes it unlikely that the testicular mass resulting from the angiosarcoma preceded the hydrocele, unless the testicular mass was present and went unnoticed by the patient for well over 7 years.

Three of the 5 cases of primary testicular angiosarcoma were associated with the concurrent presence of multilocular cystic areas diagnosed as mature teratoma in the same testicular tumor[1,3,4]. These 3 cases were mature testicular teratoma admixed with primary testicular angiosarcoma as a malignant component within the testis. These 3 cases represent heterologous sarcomatous differentiation in a testicular germ cell neoplasm. Therefore, it seems there are two groups of testicular angiosarcomas: one group (3 of the 5 cases) that occurs in young people that represent a malignancy derived from teratoma; and a second group (2 of the 5 cases) that occurs in the elderly and is not associated with germ cell neoplasm, but may be associated with chronic hydrocele. The current WHO terminology for the first group is somatic-type malignancy from teratoma or teratoma with malignant transformation. The presence of angiosarcoma as a component of testicular teratoma is extremely rare [1,3,4], and may be misdiagnosed as embryonal carcinoma [4]. The most frequent malignant components associated with testicular germ cell tumors are sarcomas, of which rhabdomyosarcoma is the most common subtype [17]. Two cases of therapy-related angiosarcoma of the retroperitoneum and gut have been documented in the English language medical literature in patients who were treated with radiotherapy with or without chemotherapy for testicular teratoma or seminoma [5,6]. Sahoo et al [4] recently described a rare association of angiosarcoma with teratoma in a patient who had received no treatment prior to surgery.

## Conclusion

Primary testicular angiosarcoma is an exceedingly rare entity for which the clinical and pathological parameters have yet to be adequately defined. They were not associated with antecedent radiotherapy or prior exposure to arsenic, thorium dioxide, or vinyl chloride. They appear to segregate into 2 groups; one associated with teratoma and occurring in young people, and the other occurring in the elderly and not associated with germ cell neoplasm, but may be associated with chronic hydrocele. These tumors present as sizeable painless hemorrhagic masses typically in the testicular parenchyma and epididymis. They share most of the histopathologic features of other deep soft tissue angiosarcomas, and hence may pose considerable diagnostic difficulty, particularly those with a solid pattern and epithelioid cytologic features. Immunohistochemical studies may be helpful, and more than one endothelial marker and at least one cytokeratin marker should be used to confirm the diagnosis of testicular angiosarcoma. Because of the limited follow-up, the behavior of this entity is uncertain, though the prognosis for these patients may be better than the previously thought generally poor prognosis for deep soft tissue and visceral angiosarcomas.

## Competing interests

The author(s) declare that they have no competing interests.

## Authors' contributions

HBA participated in the histopathological evaluation, performed the literature review, acquired photomicrographs and drafted the manuscript. UNMR and AVP conceived and designed the study, gave the final histopathological diagnosis and revised the manuscript for important intellectual content. All the authors read and approved the final manuscript.
